# Assessment of Prevalence and Risk Factors for Central Sensitization Related to Shoulder Osteoarthritis and Rotator Cuff Tears Using the Central Sensitization Inventory: A Cross-Sectional Study in Shoulder Surgery Patients

**DOI:** 10.3390/jcm12175633

**Published:** 2023-08-29

**Authors:** Ryosuke Iio, Tomoya Manaka, Katsumasa Nakazawa, Yoshihiro Hirakawa, Yoichi Ito, Ayako Ogura, Hiroaki Nakamura

**Affiliations:** 1 Department of Orthopaedic Surgery, Osaka City University Graduate School of Medicine, Osaka 545-8585, Japan; a08ma002@gmail.com (R.I.); v0vpeace@yahoo.co.jp (K.N.); 2 Department of Orthopaedic Surgery, Osaka Metropolitan University Graduate School of Medicine, Osaka 545-8585, Japan; yoshimpreza@gmail.com (Y.H.); hnakamura@omu.ac.jp (H.N.); 3 Ito Clinic, Osaka Shoulder Center, Osaka 580-0016, Japan; yito@omu.ac.jp (Y.I.); a_ogura_mail@yahoo.co.jp (A.O.)

**Keywords:** rotator cuff tear, shoulder osteoarthritis, central sensitization, central sensitization inventory, intractable pain

## Abstract

Shoulder disorders occasionally cause intractable pain. Central sensitization (CS) may be involved in such pain. Identifying risk factors associated with CS is crucial for effective pain control. This study aimed to determine the effects of shoulder osteoarthritis and rotator cuff tears (RCT) on CS and associated factors. This study included patients evaluated for CS using the Central Sensitization Inventory (CSI) before surgery for shoulder osteoarthritis, RCT, or cuff tear arthropathy. Patients with a CSI score of 40 or higher were defined as having CS. The relationships between glenohumeral osteoarthritis (GHOA), RCT size, and CS were statistically analyzed. Multiple regression analysis was performed to examine the factors affecting CSI scores. Subjects included 167 patients: 131 patients had RCT without GHOA, 23 had GHOA with RCT, and 13 had GHOA without RCT. The GHOA group had a significantly higher CSI score (27.5 [10.8–40.5] vs. 18.0 [10.0–27.5]) and CS prevalence (27.8% vs. 8.4%) than the RCT without GHOA group. There was no significant correlation between RCT size and CSI scores. Multiple regression analysis showed that female sex, severe pain, and long pain duration were associated with higher CSI scores. Considering the risk factors for CS might be helpful in shoulder treatment.

## 1. Introduction

The pathophysiology of shoulder pain due to shoulder diseases is diverse and includes tendonitis, rotator cuff tears, and osteoarthritis [1]. However, intractable pain that cannot be explained by structural changes, such as rotator cuff tears, articular cartilage wear, or synovitis, may occur. That kind of pain does not disappear simply by surgically resolving structural problems, such as rotator cuff repair, joint replacement, or debridement, leading to prolonged postoperative pain. Shoulder pain can be attributed to multiple factors, including social and psychological factors [2,3] and structural factors. Furthermore, central sensitization (CS) is associated with intractable pain. Central sensitization is defined as the “Increased responsiveness of nociceptive neurons in the central nervous system to their normal or subthreshold afferent input” by the International Association for the Study of Pain. CS causes a change in the pain threshold, making the patient feel pain more easily, leading to prolonged pain and difficulty in pain control [4]. Such chronic and intractable pain increases the socioeconomic burden due to decreased worker productivity and increased medical costs, and these costs can be greater than those of heart disease, cancer, and diabetes [5]. Therefore, the implications of central sensitization might extend beyond individual patients to become critical socioeconomic problems for communities and entire nations. Evaluation of central sensitization includes the quantitative sensory testing protocol established by the German Research Network on Neuropathic Pain [6,7] and functional magnetic resonance imaging [8]. However, these methods are time-consuming and inconvenient to perform in routine practice. On the other hand, the Central Sensitization Inventory (CSI), one of the assessment methods for CS developed in 2011, can be performed easily using a self-administered questionnaire in daily practice [9]. The Japanese version of the CSI, widely used as an evaluation method for CS, was validated [10,11]. In the field of orthopedics, central sensitization has been evaluated in the spine [12,13], hip [14,15,16], and knee joints [17,18,19], and the risk factors for CS and its effect on postoperative pain have been reported [18,19,20]. An association between osteoarthritis (OA) and CS has been reported in knee joints [21], and women are more likely to suffer from CS [22].

Although there are some reports on central sensitization of the shoulder [23,24,25], there are no reports on rotator cuff tears, shoulder osteoarthritis, and central sensitization, which account for many patients undergoing surgery for shoulder disorders. Identifying the factors related to rotator cuff tears, shoulder OA, and CS may clarify how to manage patients with persistent postoperative pain despite a surgical repair, reconstruction, or replacement to resolve structural problems. Understanding the frequency of CS and its risk factors for shoulder diseases is critical for controlling shoulder pain.

Based on previous studies implicating central sensitization in other musculoskeletal diseases [12,13,14,15,16,17,18,19], and OA [21] and female sex [22] as risk factors for central sensitization, we hypothesized that central sensitization occurs in shoulder disease as in other musculoskeletal diseases and that glenohumeral osteoarthritis (GHOA) and female sex may be risk factors for central sensitization. Therefore, the purpose of this study was to clarify the prevalence of central sensitization and its risk factors in shoulder surgery patients for OA and rotator cuff tears.

## 2. Materials and Methods

### 2.1. Patients

**Figure 1:**
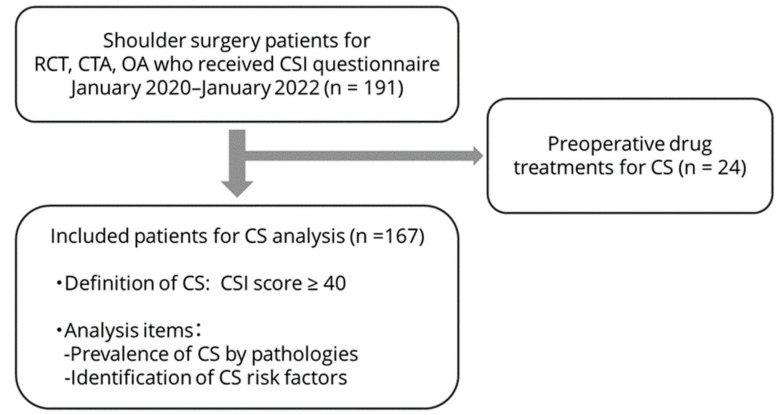
Inclusion and exclusion criteria and analysis protocol for this study. We recruited shoulder surgery patients to take the CSI questionnaire preoperatively. Patients already receiving drug treatment for CS were excluded. Prevalence and risk factors for CS in shoulder surgery patients were evaluated. RCT: rotator cuff tear, CTA: cuff tear arthropathy, OA: osteoarthritis, CSI: central sensitization inventory.

This retrospective cross-sectional study included patients who underwent unilateral shoulder surgery between January 2020 and January 2022 for rotator cuff tears, cuff tear arthropathy, or shoulder OA. A Japanese version of the CSI record was obtained preoperatively to assess CS. Patients who had received preoperative drug treatments for CS, such as tramadol, duloxetine, and pregabalin, were excluded. Details of this study protocol are shown in Figure 1. The applicable institutional review board approved this study (Approval No. 2021-266, 2021-277), and informed consent was obtained from all patients.

### 2.2. Evaluation of Central Sensitization

CS was evaluated using Part A of the Japanese version of the CSI, and a total score of 40 or higher was defined as CS. CS severity was classified into the following five categories based on CSI scores: subclinical (0–29), mild (30–39), moderate (40–49), severe (50–59), and extreme (60–100) [26]. To investigate the association between shoulder OA and CS, we assessed the differences in the incidence of CS and CSI scores between patients with and without GHOA. Additionally, we subdivided patients with GHOA by the presence or absence of rotator cuff tears to examine the effect of the presence or absence of rotator cuff tears on CS in patients with GHOA. Furthermore, the relationship between rotator cuff tear size and CS was evaluated to determine the influence of rotator cuff tears on CS in patients without GHOA. To identify the factors associated with the CSI score, multiple linear regression analysis was performed using age, sex, range of motion (flexion, external rotation, and internal rotation), pain intensity, duration of pain, history of surgery or trauma, and presence of GHOA and rotator cuff tears as explanatory variables. Internal rotation was assessed using the Constant score, and pain intensity was evaluated using the visual analog scale (VAS). Patient background and clinical data were collected using REDCap (Vanderbilt University, Nashville, TN, USA).

### 2.3. Statistical Analysis

Results that follow a normal distribution are presented as mean ± standard deviation, and data that do not follow a normal distribution are shown as median [interquartile range]. Student’s *t*-test was used to compare the two groups following a normal distribution, and the Mann-Whitney U test was used for those not following a normal distribution. Categorical variables were evaluated using the chi-squared test. The association between rotator cuff tear size and CS was evaluated in the groups according to tear size using the Kruskal-Wallis test, followed by the post hoc Steel-Dwass test. Multiple linear regression analysis with the CSI score as the objective variable was performed as a multivariable analysis to identify risk factors for CS. All statistical analyses were conducted using R (version 3.6.1; R Foundation for Statistical Computing, Vienna, Austria) with a significance level of *p* < 0.05. Because this was a retrospective study, sample size calculations were not performed. However, a post hoc power analysis using G*Power software (version 3.1.9.7; University of Dusseldorf, Dusseldorf, Germany), which is a statistical power analysis software tool, showed a power of 0.82 with an alpha error of 0.05 for detecting a significant difference in CSI values between patients with and without OA, a finding which is the primary outcome of this study.

## 3. Results

### 3.1. Demographic Data

This study included 167 patients (84 males and 83 females) with a median age of 70.0 [63.0–75.0] years; 131 patients (78.4%) had rotator cuff tears without GHOA, 23 (13.8%) had GHOA with rotator cuff tears, and 13 (7.8%) had GHOA without rotator cuff tears. The patients’ demographic data are shown in detail in Table 1.

### 3.2. Incidence and Severity of Central Sensitization

The median CSI score was 18.0 [10.0–32.0], and the incidence of CS with a CSI score of 40 or more was 12.6% in all patients (Table 1). Moderate, severe, and extreme severity accounted for 8.4%, 3.9%, and 1.2% of all cases, respectively (Table 2).

### 3.3. Association of Central Sensitization with Glenohumeral Osteoarthritis and Rotator Cuff Tears

The results of the association between GHOA and CS are shown in Table 3. The CSI scores were significantly higher in the GHOA group (with rotator cuff tear and without rotator cuff tear) (27.5 [10.8–40.5]) than in the non-OA group (with rotator cuff tear) (18.0 [10.0–27.5]) (*p* = 0.043). The incidence of CS with a CSI score of 40 or higher was considerably higher in the group with GHOA (with rotator cuff tear and without rotator cuff tear) (27.8%) compared to the group without GHOA (with rotator cuff tear) (8.4%) (*p* = 0.004). Patients with GHOA were significantly older than those without it (*p* = 0.001). Patients with GHOA tended to have longer pain duration than those without GHOA, which was insignificant due to wide variability (0.7 [0.3–2.0] vs. 0.5 [0.3–1.0], *p* = 0.24).

Furthermore, since it is necessary to assess whether the presence or absence of rotator cuff tears affects CS among patients with GHOA, we further subdivided the GHOA group by the presence or absence of rotator cuff tears to examine the effect of rotator cuff tears on central sensitization. The results regarding the effect of central sensitization with and without rotator cuff tears in GHOA patients are shown in Table 4. The presence or absence of rotator cuff tears in patients with GHOA did not significantly differ in the incidence of central sensitization or CSI values. However, OA patients with rotator cuff tears were significantly older than those without rotator cuff tears (*p* = 0.004). The other patients’ backgrounds were not different between them.

The results of the associations between rotator cuff tears and CS are shown in Table 5. Comparisons of rotator cuff tear size and CS among patients without GHOA revealed no significant differences in rotator cuff tear size, CSI score, or CS incidence.

Patients with large or massive tears were significantly older than those with partial-thickness and small/medium-sized tears. The other patients’ characteristics were not different between them.

### 3.4. Factors Associated with Central Sensitization

The results of the multiple linear regression analysis are presented in Table 6. Sex, pain VAS score, and pain duration were independent factors associated with CSI scores. Female sex, high pain VAS scores (mm), and long pain duration (year) were risk factors for increased CSI scores with coefficients of 5.6, 0.14, and 0.90, respectively.

## 4. Discussion

In this study, we hypothesized that patients with GHOA, especially women, are more likely to develop CS and undergo shoulder surgery. Supporting this hypothesis, patients with GHOA had significantly higher CSI scores and a higher incidence of central sensitization than those without GHOA. On the other hand, the presence or absence of rotator cuff tears in patients with GHOA did not significantly differ in the incidence of CS or CSI scores, nor did the incidence of CS or CSI scores by rotator cuff tear size without GHOA. Multivariable analysis also revealed that female sex, severe pain, and longer pain duration were independent risk factors for CS in shoulder surgery patients for shoulder OA and rotator cuff tears.

In this current study on shoulder surgery, patients with rotator cuff tears and GHOA, the incidence of CS was 12.6%. The incidence of CS by disease was 8.4% in patients with rotator cuff tears without OA and 27.8% in patients with OA. CS is defined by the International Association for the Study of Pain as the “Increased responsiveness of nociceptive neurons in the central nervous system to their normal or subthreshold afferent input” and is a condition of increased pain sensitivity due to changes in the pain threshold [4]. The characteristics of CS are reported as “Pain experience disproportionate to the nature and extent of injury or pathology”, “Diffuse pain distribution, allodynia, and hyperalgesia”, and “Hypersensitivity of senses unrelated to the musculoskeletal system [27]”. The CSI was developed in 2011 as an index to evaluate CS [9], and it is widely used to assess CS [11]. A Japanese version of the CSI has also been developed and validated [10]. Various diseases are associated with CS, including fibromyalgia, headaches, temporomandibular disorders, and visceral pain hypersensitivity syndromes [28]. Previous reports showed that CS affects musculoskeletal diseases with intractable pain and poor surgical outcomes in back pain [12,13], lateral epicondylitis [29], carpal tunnel syndrome [30], knee OA [18,19,21], and hip OA [14,15,16]. CS negatively affects chronic shoulder pain [24], frozen shoulder [25], and impingement syndrome [23,31].

Regarding the effect of CS on postoperative outcomes, the clinical outcomes of TKA in patients with preoperative CS were poorer than those in patients without CS [18,19]. Furthermore, perioperative therapeutic interventions for patients with preoperative CS improve postoperative TKA outcomes [32]. This study revealed that shoulder diseases such as rotator cuff tears and GHOA, as well as other musculoskeletal disorders, affected a certain number of patients with CS. A high percentage of patients with GHOA had central sensitization (27.8%). CS is associated with prolonged pain after TKA or THA [18,19,20], and persistent postoperative pain also occurs in shoulder disorders such as impingement syndrome [23]. Furthermore, a report has demonstrated that perioperative intervention for CS improves postoperative outcomes [32], identifying that the presence or absence of preoperative CS may prevent prolonged postoperative pain and poor clinical outcomes in shoulder surgery. Further studies are required to determine the influence of preoperative CS on postoperative outcomes.

This study revealed that patients with GHOA had significantly higher CS incidence rates and CSI scores than those without GHOA. Patients with GHOA had a longer duration of pain, although the difference was not significant, with considerable variation. There was no significant difference in the CSI score according to the rotator cuff tear size. Regarding the relationship between OA and CS, patients with knee OA were more likely to experience pain sensitization than those without OA. The mechanism of CS caused by OA is related to excessive ascending nociceptive signaling and insufficient descending inhibitory signaling, which are maintained by peripheral nociceptive input from the OA joint [33], suggesting that OA may be one of the causes of CS. In contrast, there are no reports of rotator cuff tears involving CS, and pain does not correlate with tear size [34,35]. This study showed significantly higher CS in the group with GHOA, and that OA of other joints caused CS, as previous reports have shown [17], suggesting that GHOA may also cause CS. Furthermore, the presence or absence of rotator cuff tears did not affect the incidence of CS or the CSI score in patients with GHOA, suggesting that the presence of GHOA affects CS regardless of the presence or absence of rotator cuff tears. On the other hand, rotator cuff tear size was not associated with CS in this study, and pain was not correlated with tear size. The association between rotator cuff tear size and pain intensity in the present study is similar to that reported in previous studies; the presence of rotator cuff tear and differences in that size may not affect CS.

Multivariable analysis undertaken in the current study to identify independent risk factors for CS revealed that sex, pain intensity, and duration of disease are independent factors that contribute to the CSI score. With regard to CS and sex, women are at higher risk of CS than men [22]. In addition, there was a significant association between pain duration and CS in knee OA [21]. In this study, female sex, pain intensity, and pain duration were independent risk factors for CS in patients with shoulder disease, rotator cuff tears, or GHOA. These risk factors are consistent with those reported in previous studies on CS. Although patients with GHOA in this study had a significantly higher prevalence rate of CS than those without GHOA, multivariable analysis showed that GHOA was not an independent factor contributing to the CSI score. However, patients with GHOA tended to have a longer pain duration than those without GHOA, a factor which might affect CS. In the treatment of shoulder disorders with rotator cuff tears or GHOA, CS should be considered in patients with risk factors for increased CSI scores: severe pain, long duration of pain, and female sex. Of these factors, female sex, with its coefficient of 5.6 in multivariate analysis, should be given particular attention, as its coefficient is higher than that of pain intensity or duration.

This study had several limitations. First, because this was a retrospective study, data collection on patient background, including patients’ physical activity levels, lifestyle habits, and comorbidities, was limited. A previous report suggests that lifestyle factors, such as sleep deprivation, stress, diet, smoking, and physical inactivity, should be considered in understanding central sensitization [36]. Another report shows that managing chronic pain with central sensitization requires addressing comorbidities such as insomnia and obesity and lifestyle factors such as stress, physical inactivity, and unhealthy diet [37]. Therefore, we should consider patients’ lifestyles and comorbidities to clarify the potential factors of CS in shoulder joint disease in future studies. Second, only patients who underwent surgery were evaluated, which could have led to a patient selection bias. Furthermore, this study included only patients who underwent surgery for rotator cuff tears, rotator cuff tear arthropathy, or shoulder OA, but not for other shoulder disorders or patients who did not undergo surgery. Therefore, future studies are needed to clarify the effects of CS in patients with shoulder diseases, including those with other conditions or those undergoing conservative treatment. Finally, the relationship between preoperative CS and postoperative outcomes was not examined in this study. Some reports have shown that high preoperative CSI is associated with poor postoperative outcomes in knee [18,19] and hip OA [20]. Therefore, we should examine the effects of preoperative CS and CSI scores on postoperative clinical outcomes in shoulder joint disease. A report showed that perioperative intervention by drug treatments for CS improved postoperative clinical outcomes [28]. Hence, the necessity for CS intervention in shoulder diseases should also be investigated in the future.

## 5. Conclusions

In shoulder surgery patients for shoulder OA and rotator cuff tear, the incidence of CS occurred at 12.6% in this study. Regarding the incidence of CS by pathological conditions, 27.8% of the patients with GHOA had CS in this study, a significantly higher percentage than rotator cuff tear patients without GHOA (8.4%). Rotator cuff tear was not related to the CS incidence and CSI score in GHOA patients. Furthermore, female sex, pain intensity, and duration of pain are independent factors that contribute to high CSI scores and may be risk factors for the incidence of CS, indicating that these risk factors should be considered when treating pain due to shoulder disorders.

## Figures and Tables

**Table 1:** Demographic data.

	All Patients (n = 167)
Age (years)	70.0 [63.0–75.0]
Sex—Male, n (*%*)	84 (50.3)
Sex—Female, n (*%*)	83 (49.7)
Affected side—Right, n (*%*)	102 (61.1)
Affected side—Left, n (*%*)	65 (38.9)
CSI	18.0 [10.0–32.0]
CSI < 40, n (%)	146 (87.4)
CSI ≥ 40, n (%)	21 (12.6)
Pain VAS (mm)	35.0 [11.0–64.0]
Pain duration (year)	0.5 [0.3–1.2]
Active flexion (degrees)	125.0 [80.0–150.0]
Active abduction (degrees)	100.0 [60.0–152.5]
Active external rotation (degrees)	35.0 [20.0–52.5]
Active internal rotation (Points, based on Constant score)	6.0 [4.0–8.0]
Rotator cuff tear without osteoarthritis of GH joint, n (%)	131 (78.4)
Osteoarthritis of GH joint with rotator cuff tear, n (%)	23 (13.8)
Osteoarthritis of GH joint without rotator cuff tear, n (%)	13 (7.8)
History of trauma, n (%)	40 (24.0)
History of shoulder surgery	20 (12.0)

Data are shown as median [interquartile range] unless otherwise noted. VAS: visual analog scale, GH: glenohumeral, CSI: central sensitization inventory.

**Table 2:** Classification of central sensitization severity.

CSI Score	n (%)
Subclinical (0–29)	120 (71.6)
Mild (30–39)	26 (15.6)
Moderate (40–49)	14 (8.4)
Severe (50–59)	5 (3.9)
Extreme (60–100)	2 (1.2)

CSI: central sensitization inventory.

**Table 3:** Evaluation of central sensitization patients’ characteristics with or without osteoarthritis.

	OA Patients (with RCT and without RCT) (n = 36)	Non-OA Patients (with RCT) (n = 131)	*p*-Value
CSI	27.5 [10.8–40.5]	18.0 [10.0–27.5]	0.043 *
CSI ≥ 40, n (%)	10 (27.8)	11 (8.4)	0.004 *
Age (years)	74.5 [68.8–79.0]	69.0 [60.5–74.0]	0.001 *
Sex—Male/Female, n	15/21	69/62	0.26
Affected side— Right/Left, n	25/11	77/54	0.34
Pain VAS (mm)	32.5 [9.8–63.5]	74.5 [13.0–62.5]	0.52
Pain duration (year)	0.7 [0.3–2.0]	0.5 [0.3–1.0]	0.24

Data are indicated as median [interquartile range] unless otherwise noted. CSI: central sensitization inventory, VAS: visual analog scale, OA: osteoarthritis, RCT: rotator cuff tear. * Statistically significant (*p* < 0.05).

**Table 4:** Evaluation of central sensitization in OA patients with or without rotator cuff tears.

	OA Patients with RCT (n = 23)	OA Patients without RCT (n = 13)	*p*-Value
CSI	25.7 ± 16.9	31.2 ± 21.5	0.41
CSI ≥ 40, n (%)	6 (26.1)	4 (30.8)	1.0
Age (years)	69.0 [68.0–71.0]	76.0 [73.5–79.0]	0.007 *
Sex—Male/Female, n	10/13	5/8	1.0
Affected side— Right/Left, n	16/7	9/4	1.0
Pain VAS (mm)	25.0 [9.5–59.0]	40.0 [10.0–70.0]	0.79
Pain duration (year)	1.0 [0.2–2.3]	0.6 [0.4–1.3]	0.54

Parametric data are indicated as mean ± standard deviation, and nonparametric data are shown as median [interquartile range] unless otherwise noted. CSI: central sensitization inventory, VAS: visual analog scale, OA: osteoarthritis, RCT: rotator cuff tear. * Statistically significant (*p* < 0.05).

**Table 5:** Comparison of central sensitization and patients’ characteristics between rotator cuff tear sizes without osteoarthritis.

	Partial-Thickness Tear (n = 19)	S and M Size Tear (n = 60)	L and Ma Size Tear (n = 52)	*p*-Value
CSI	25.1 ± 13.5	18.4 ± 11.2	19.7 ± 13.6	0.12
CSI ≥ 40, n (%)	4 (21.1)	2 (3.3)	5 (9.6)	0.038 *
Age (years)	62.0 [58.5–65.5]	67.5 [55.8–73.0]	72.5 [68.0–77.0]	<0.001 *
Sex— Male/Female, n	10/9	30/30	29/23	0.83
Affected side— Right/Left, n	9/10	39/21	29/23	0.34
Pain VAS (mm)	40.0 [26.0–71.5]	35.0 [14.3–59.3]	35.5 [10.8–61.3]	0.61
Pain duration (year)	0.4 [0.3–0.8]	0.6 [0.3–1.0]	0.4 [0.3–1.0]	0.64
Post hoc test (Bonferroni) regarding CSI ≥ 40				
Partial-thickness tear and S and M size tear	Partial-thickness tear and L and Ma size tear		S and M size tear and L and Ma size tear	
0.080	0.71		0.74	
Post hoc test (Steel-Dwass test) regarding age				
Partial-thickness tear and S and M size tear	Partial-thickness tear and L and Ma size tear		S and M size tear and L and Ma size tear	
0.21	<0.001 *		<0.001 *	

Data following normal distribution are indicated as mean ± standard deviation, and data not following normal distribution are shown as median [interquartile range] unless otherwise noted. CSI: central sensitization inventory, VAS: visual analog scale, S: small, M: medium, L: large, Ma: massive. * Statistically significant (*p* < 0.05).

**Table 6:** Multiple linear regression analysis for the central sensitization inventory score.

Risk Factor	Coefficients (95% CI)	SE	T-Value	*p*-Value
Age (years)	0.11 (−0.10–0.32)	0.11	1.0	0.31
Sex—Female	5.6 (1.6–9.6)	2.0	2.7	0.007 *
Pain VAS (mm)	0.14 (0.066–0.22)	0.038	3.7	<0.001 *
Pain duration (year)	0.90 (0.019–1.8)	0.45	2.0	0.045 *
Active flexion (degrees)	−0.048 (−0.13–0.004)	0.033	−1.9	0.065
Active external rotation (degrees)	−0.046 (−0.15–0.059)	0.053	−0.87	0.39
Active internal rotation (Points, based on Constant score)	0.33 (−0.67–1.3)	0.50	0.65	0.52
Rotator cuff tear	−1.4 (−11–8.7)	5.1	−0.27	0.79
Osteoarthritis of GH joint	1.5 (−5.2–8.2)	3.8	0.45	0.65
History of trauma	−2.2 (−7.0–2.5)	2.4	−0.93	0.35
History of shoulder surgery	−3.3 (−11–4.7)	4.0	−0.82	0.41

CI, confidence interval; SE, standard error; VAS, visual analog scale; GH, glenohumeral. * Statistically significant (*p* < 0.05).

## Data Availability

The datasets used and analyzed in this current study are available from the corresponding author upon reasonable request.
